# Supplementary material for: Determining the worldwide epidemiology of surgical site infections after gastrointestinal resection surgery: protocol for a multicentre, international, prospective cohort study (GlobalSurg 2)
Source: BMJ Open. 2017 Jul 21;7(7):e012150. doi: 10.1136/bmjopen-2016-012150 (PMC5577891; doi:10.1136/bmjopen-2016-012150)
Supplement: Supplementary file 1 [file bmjopen-2016-012150supp001.pdf]

South East Scotland Research Ethics Service

Waverley Gate
2-4 Waterloo Place
Edinburgh
EH1 3EG

NHS Lothian

Name: Stuart Fergusson

Date: 16/10/2015
Your Ref:
Our Ref: NR/1510AB5
Enquiries to: Alex Bailey
Direct Line: 0131 465 5679
Email: alex.bailey@nhslothian.scot.nhs.uk

Dear Stuart,

Project Title: Determining the worldwide epidemiology of surgical site infections after gastrointestinal surgery

You have sought advice from the South East Scotland Research Ethics Service on the above project. This has been considered by the Scientific Officer and you are advised that, based on the submitted documentation (email correspondence and GS2 protocol v7_4_SJF), it does not need NHS ethical review under the terms of the Governance Arrangements for Research Ethics Committees (A Harmonised Edition).

The advice is based on the following:

- *The project is an audit limited to using data obtained as part of usual care, but note the requirement for Caldicott Guardian approval for the use or transfer of person-identifiable information within or from an organisation*

If the project is considered to be health-related research you will require a sponsor and ethical approval as outlined in The Research Governance Framework for Health and Community Care. You may wish to contact your employer or professional body to arrange this. You may also require NHS management permission (R&D approval). You should contact the relevant NHS R&D departments to organise this.

For projects that are not research and will be conducted within the NHS you should contact the relevant local clinical governance team who will inform you of the relevant governance procedures required before the project commences.

This letter should not be interpreted as giving a form of ethical approval or any endorsement of the project, but it may be provided to a journal or other body as evidence that NHS ethical approval is not required. However, if you, your sponsor/funder feel that the project requires ethical review by an NHS REC, please write setting out your reasons and we will be pleased to consider further. You should retain a copy of this letter with your project file as evidence that you have sought advice from the South East Scotland Research Ethics Service.

Yours sincerely,

Alex Bailey

Alex Bailey
Scientific Officer
South East Scotland Research Ethics Service

INVESTORS IN PEOPLE
POSITIVE ABOUT DISABLED PEOPLE
Healthy Working Lives

Headquarters
Waverley Gate, 2-4 Waterloo Place
Edinburgh EH1 3EG
Chair: Mr Brian Houston
Chief Executive: Tim Davison
Lothian NHS Board is the common name of Lothian Health Board

Differentiating clinical audit, service evaluation, research and usual practice/surveillance work in public health

RESEARCH	SERVICE EVALUATION*	CLINICAL AUDIT	SURVEILLANCE	USUAL PRACTICE (in public health)
The attempt to derive generalizable new knowledge including studies that aim to generate hypotheses as well as studies that aim to test them.	Designed and conducted solely to define or judge current care.	Designed and conducted to produce information to inform delivery of best care.	Designed to manage outbreak and help the public by identifying and understanding risks associated.	Designed to investigate outbreak or incident to help in disease control and prevention.
Quantitative research – designed to test a hypothesis. Qualitative research – identifies/explores themes following established methodology.	Designed to answer: "What standard does this service achieve?"	Designed to answer: "Does this service reach a predetermined standard?"	Designed to answer: "What is the cause of this outbreak?"	Designed to answer: "What is the cause of this outbreak?" and treat.
Addresses clearly defined questions, aims and objectives.	Measures current service without reference to a standard.	Measures against a standard.	Systematic, statistical methods to allow timely public health action.	Systematic, statistical methods may be used.
Quantitative research – may involve evaluating or comparing interventions, particularly new ones. Qualitative research – usually involves studying how interventions and relationships are experienced.	Involves an intervention in use only. The choice of treatment is that of the clinician and patient according to guidance, professional standards and/or patient preference.	Involves an intervention in use only. The choice of treatment is that of the clinician and patient according to guidance, professional standards and/or patient preference.	May involve collecting personal data and samples with the intent to manage the incident.	Any choice of treatment is based on clinical best evidence or professional consensus.
Usually involves collecting data that are additional to those for routine care but may include data collected routinely. May involve treatments, samples or investigations additional to routine care.	Usually involves analysis of existing data but may include administration of interview or questionnaire.	Usually involves analysis of existing data but may include administration of simple interview or questionnaire.	May involve analysis of existing data or administration of interview or questionnaire to those exposed.	May involve administration of interview or questionnaire to those exposed.
Quantitative research – study design may involve allocating patients to intervention groups. Qualitative research – uses a clearly defined sampling framework underpinned by conceptual or theoretical justifications.	No allocation to intervention: the health professional and patient have chosen intervention before service evaluation.	No allocation to intervention: the health professional and patient have chosen intervention before audit.	Does not involve an intervention.	May involve allocation to control group to assess risk and identify source of incident but treatment unaffected.
May involve randomisation.	No randomisation.	No randomisation.	No randomisation.	May involve randomisation but not for treatment.
Normally requires REC review. Refer to www.nres.npsa.nhs.uk/applications/apply/ for more information.	Does not require REC review.	Does not require REC review.	Does not require REC review.	Does not require REC review.

* Service development and quality improvement may fall into this category.

Headquarters
Waverley Gate, 2-4 Waterloo Place
Edinburgh EH1 3EG
Chair: Mr Brian Houston
Chief Executive: Tim Davison
Lothian NHS Board is the common name of Lothian Health Board
