# Supplementary material for: Determining the worldwide epidemiology of surgical site infections after gastrointestinal resection surgery: protocol for a multicentre, international, prospective cohort study (GlobalSurg 2)
Source: BMJ Open. 2017 Jul 21;7(7):e012150. doi: 10.1136/bmjopen-2016-012150 (PMC5577891; doi:10.1136/bmjopen-2016-012150)
Supplement: Author's manuscript [file bmjopen-2016-012150.draft_revisions.pdf]

BMJ Open

Determining the worldwide epidemiology of Surgical Site Infections after Gastrointestinal Resection Surgery: a multicentre, international, prospective cohort study (GlobalSurg 2)

Journal:	*BMJ Open*
Manuscript ID	bmjopen-2016-012150
Article Type:	Protocol
Date Submitted by the Author:	07-Jun-2016
Complete List of Authors:	Collaborative, GlobalSurg
**Primary Subject Heading**:	Surgery
Secondary Subject Heading:	Infectious diseases
Keywords:	Globalsurgery, Surgical site Infection, Collaborative

SCHOLARONE™
Manuscripts

For peer review only

Determining the worldwide epidemiology of Surgical Site Infections after Gastrointestinal Resection Surgery: a multicentre, international, prospective cohort study (GlobalSurg 2)

Author: GlobalSurg Collaborative

***Correspondence*:**

Mr Aneel Bhangu MBChB MRCS PhD, Academic Department of Surgery
Room 29, 4th Floor, University of Birmingham, Birmingham, B15 2TH, UK
Email: aneelbhangu@doctors.org.uk , collaborate@globalsurg.org

Declarations

Running title:	GlobalSurg 2 Project
Funding/Disclosure:	This project has received support from the British Medical Association and the Association of Surgeons in Training. These organisations have not been involved in the drafting of this protocol or review of this manuscript.
Abstract word count:	177
Word count:	2807 (excluding abstract, tables and references)
Key words:	Global Surgery, Surgical Site Infection

Authors' contributions: All authors contributed significantly to study conception, protocol development, manuscript writing/editing and agreement for submission.

For peer review only - http://bmjopen.bmj.com/site/about/guidelines.xhtml

ABSTRACT

Introduction: Surgical site infection (SSI) is the most common complication following major gastrointestinal surgery, affecting between 25-40% of patients. The rate of SSI doubles from low-income to high-income settings, persisting after risk adjustment. The relative impact of antibiotic resistant organisms and the effectiveness of antibiotic prophylaxis globally are unknown. This study aims to determine SSI rates following gastrointestinal surgery across worldwide hospital settings.

Methods and analysis: This multicentre, international, prospective cohort study will be undertaken by any hospital providing emergency or elective gastroenterological surgical services. Centres will collect observational data on consecutive patients undergoing emergency or elective gastrointestinal resection, cholecystectomy or appendicectomy during a six-month period. The primary outcome is the incidence of SSI with secondary outcomes describing the organisms causing SSIs, including their antibiotic susceptibility, and the microbiological tests used to identify them.

Ethics and dissemination: This project will not affect clinical practice and has been classified as clinical audit following research ethics review. The protocol will be disseminated through the international GlobalSurg network.

Trial Registration Number: This study has been registered with ClinicalTrials.gov (identifier: NCT02662231).

For peer review only

For peer review only - http://bmjopen.bmj.com/site/about/guidelines.xhtml

Strengths and Limitations

- This will be the first international, multicentre, prospective study to assess the incidence of surgical site infections (SSI) for patients undergoing elective and emergency major gastrointestinal surgery. It will be delivered across the GlobalSurg collaborative, an international network of grass-roots surgeons from 92 countries.

- The collaborative methodology adopted, as described elsewhere [1], allows for large volume, high quality data collection. Short, 2-week study periods, avoid overburdening high-volume and low-resource centres that may otherwise be unable to participate in such projects. A 6-month data collection window provides maximal opportunity for collaborator involvement, and standardises for seasonal variation in outcomes.

- Previous studies have had varying subjective definitions of SSI; this study uses standardised definitions from the Centre for Disease Control and Prevention (CDC) [2], which are widely implemented and internationally validated.

- Due to the observational nature of the study, it will not be possible to determine a causative link between risk factors and SSI. Observations of the incidence of SSI and identification of risk factors across different Human Development Index settings will therefore be hypothesis generating only. This will aid to design and power future global surgery randomised controlled trials.

- Within the confines of this study it is not possible to apply strict data monitoring and quality regulations, such as those applied in randomised controlled studies. We have therefore developed a mixed-methods quantitative and qualitative validation process for our primary outcome measure, and case ascertainment rate, which will be applied to a sample of representative centres.

For peer review only - http://bmjopen.bmj.com/site/about/guidelines.xhtml

Introduction

The burden of surgical disease in low and middle-income countries (LMICs) is growing [3]. Specific programmes have aimed to highlight the unmet need for safe surgery and anaesthesia as part of the global health agenda [4]. The Lancet Commission on Global Surgery outlined six core indicators for the assessment of global surgical systems, including the postoperative mortality rate (POMR) [5]. While clearly important, mortality only affects 1-4% of all patients [6]. For major gastrointestinal surgery, quantifying POMR alone neglects the burden of postoperative morbidity, which affects a greater proportion of patients [4]. Other relevant markers of postoperative outcome are also needed to determine the success of surgery in the majority of patients who will survive.

Surgical site infection (SSI) is the most common complication following major gastrointestinal surgery [7], affecting between 25-40% of patients after midline laparotomy in high-income settings [8,9]. SSI is implicated in one-third of postoperative deaths and accounts for 8% of all deaths caused by a nosocomial infection [10–12]. Furthermore, SSIs cause pain and discomfort, increase hospital stay and put patients at greater risk of secondary infectious complications. This has an important economic impact with an attributable cost in the UK of £30 million per year [13].

Our 2014/15 prospective, observational cohort study (GlobalSurg 1 [14]) included 10,475 patients from 58 countries. It showed that the incidence of SSI more than doubled from high (7.4%), to middle (14.4%), to low (20.0%) income countries. This persisted after multivariable risk adjustment for patient and hospital confounders (middle income: odds ratio 1.96 [1.63-2.32] and low income: 2.06 [1.67-2.57]). In the most contaminated procedures, one in three patients from LMICs suffered an SSI. Dirty surgery doubled in low-income countries (29.7% versus 16.6% in high-income settings), which was in turn associated with doubling of SSI (34.5% low-income versus 15.4% high-income). However, SSI was assessed as a secondary outcome measure as part of that study, lacking validity and requiring external validation.

Antibiotic resistant organisms are now prevalent worldwide and a focus of interest for policy leaders and global health advocates [15]. In high-income settings, methicillin resistant *S. Aureus* accounts for up to 50% of *S. Aureus* infections [16]. While many hospitals in low income settings are not able to routinely perform surveillance of

For peer review only - http://bmjopen.bmj.com/site/about/guidelines.xhtml

resistant patterns, yet the little evidence that does exist suggests an accelerating incidence of antibiotic-resistant SSIs [17]. Patients with antibiotic resistant infections have a higher risk of mortality, morbidity and require more healthcare resources [18]. Currently, few data exist describing the international epidemiology of SSIs, the causative organisms or incidence of antimicrobial drug-resistance. Investigating the diagnosis and treatment of SSIs is an urgent global health priority, with important implications for patients, clinicians and policy makers.

Primary Aims

The primary aim is to determine the incidence of SSI following elective and emergency gastrointestinal surgery across low, middle and high Human Development Index (HDI) countries.

Secondary Aims

The secondary aims include describing the organisms causing SSIs, the incidence of antibiotic resistant pathogens and the microbiological tests used to identify them. The impact of the method of 30-day follow-up on these outcomes, where performed, will also be analysed.

For peer review only

For peer review only - http://bmjopen.bmj.com/site/about/guidelines.xhtml

Methods

Study Design

This is a multicentre, international, prospective, observational cohort study of all consecutive patients undergoing elective or emergency gastrointestinal surgery over a 14-day period. Individual collaborators are free to choose any 14-day period within the six-month study period to collect data. The study will run from the 4th of January 2016 to 31st July (with 30-day follow up of the last data collection period finishing on 30th August 2016). This 'snapshot' study design is a validated model that has been delivered successfully in previous studies [8,1,19].

The GlobalSurg Network

GlobalSurg (http://globalsurg.org/) is an international research collaborative, fostering regional, national and international surgical networks. The collaborative model used has previously been described elsewhere [20] and has already facilitated one multicentre, international, prospective cohort study [21]. GlobalSurg is run by the Surgical Research Gateway (SuRG) Foundation (http://surgfoundation.org/; registered charity number 1 159 898), in collaboration with the University of Birmingham and University of Edinburgh. The objective of the charity is to advance the education of medical students and doctors in surgical science, clinical research and audit methods by promoting participation in collaborative clinical research and audit studies.

Study Setting

Any surgical unit providing emergency or elective surgery worldwide is eligible to participate. Included centres must capture all consecutive patients and ensure data collection is >95% complete. Centres with >5% missing data, when including all possible data points, will be excluded from the final analysis and removed from authorship. The minimum number of patients that must be recruited is one. Multiple teams covering different non-overlapping time periods at each hospital are encouraged.

Patient inclusion and exclusion criteria

Patients of all ages (adult and paediatric) undergoing elective, semi-elective or emergency gastrointestinal resection, cholecystectomy or appendicectomy are eligible to enter (Box 1). Any operative approach can be used. Operations with a

For peer review only - http://bmjopen.bmj.com/site/about/guidelines.xhtml

primary indication classified as vascular surgery, gynaecology, urology or transplantation should be excluded.

Emergency procedures are defined as unplanned operations and include reoperations after previous procedures. Semi-elective procedures are defined as procedures planned on the background of the patient being discharged after an emergency admission in the past and is currently admitted for surgery. Gastrointestinal resection is defined as a complete transection and removal of a segment of the oesophagus, stomach, small bowel, colon or rectum.

Each individual patient should be included once into the study. Patients who return to theatre due to complications following earlier surgery can only be included if the index procedure is not already included.

Outcome measures

The primary outcome measure is the incidence of superficial or deep incisional SSI within 30 days of surgery involving any of the operative incisions made. This measure adopts the definitions within the 2008 Centre for Disease Control (CDC) [2] definitions of SSI (Box 2). Where it is unfeasible to follow up the patients after discharge for 30 days postoperatively, in-hospital SSI incidence will be used as a proxy measure.

The secondary outcomes that will be derived from this study include the:

1. A description of the global variation in organisms causing SSI (including use of microbiological tests).
2. The incidence of antibiotic resistant pathogens detected in patients with SSI.
3. 30-day post-operative mortality rate.
4. 30-day post-operative re-intervention rate.
5. To assess how difference in SSI rates vary based on the method by which patients are followed up.

Data points

Data points relating to patient, immunosuppression status, operative method and post-operative period will be collected (Table 1). In order to maximise data completeness, a minimal data set has been designed to test only factors relevant to the outcome measures. Investigators will enter data via the secure internet-based

For peer review only - http://bmjopen.bmj.com/site/about/guidelines.xhtml

Research Electronic Data Capture (REDCap) system [22]. Anonymous patient data will be held on the system hosted by the University of Edinburgh, Scotland, UK.

Investigators

The study will be undertaken by investigators around the world who will be responsible for disseminating the protocol at their individual site, ensuring appropriate study approvals are in place and collecting and uploading data to an online REDCap database. A central study writing committee compromising of an internationally representative group of health-care professionals will be responsible for data analysis, final manuscript drafting and submission. Individuals will be required to register their unit via the REDCap system and will be required to complete two training modules prior to starting.

Countries with multiple sites will be assigned a country lead, who will be responsible for co-ordinating multiple teams across sites to ensure duplication of data does not occur. Each hospital will have a primary local investigator who will act as guarantor of data. A maximum of three local investigators can cover each two week data collection period. They will be responsible for gaining local audit, service evaluation or research ethics approval as appropriate to their institution. Investigators should also create clear mechanisms to identify and include all eligible patients, which involves daily review of operating log books, emergency admission lists and team handover lists. They should identify clear pathways to accurately collect baseline and follow up data and be proactive in identifying postoperative events within the normal limits of follow-up.

Local arrangements for follow-up may include daily review of the patient and notes during admission and before discharge in order to identify in hospital SSIs, reviewing patient status in outpatient clinics or via telephone interview at 30 days (if this normal practice), checking hospital notes (paper or electronic) and looking at handover lists. All investigators will be listed as collaborators on resulting publications.

Quality of Data

To ensure high data quality, a detailed protocol has been produced and published online. The protocol has been translated into common languages to ease investigator understanding, including Chinese (Traditional and Simplified), French, Spanish,

For peer review only - http://bmjopen.bmj.com/site/about/guidelines.xhtml

Italian, Greek, Portuguese and Arabic [23]. In addition, investigators are mandated to complete two online educational modules, one a summary of the study protocol and the other an overview of the diagnosis and classification of surgical site infection.

Data Validation

Data validation will be performed in two parts across a group of representative centres. Case completeness assessment will involve an independent investigator ascertaining the number of eligible cases within a two-week data collection period at a participating centre and comparing this to actual number of cases submitted. Data accuracy will be assessed using qualitative, semi-structured interviews with investigators to assess local data collection methods.

Statistical analysis and power calculation

Identification of hospital or surgeon-specific performance will not be reported. Variation in outcome across different contexts will be tested using the Human Development Index (HDI) [24] (a composite statistic of life expectancy, education and income indices). Following analysis, centre-specific SSI rates will be fed back to participants. Hierarchical logistic regression multivariate analysis will be used to adjust the influence of HDI on SSI rates for confounding patient and disease variables.

An appropriate sample size is difficult to calculate in this setting as there is a lack of previous study data to indicate the incidence of SSIs internationally. The previous GlobalSurg study recruited 10,745 patients using the same methodology [25].Although the inclusion criterion for this study is different, we expect to achieve a similar number of patients. Based on the findings from GlobalSurg 1, this number will provide adequate power to show a minimum difference from 7% to 10% in SSI rate between two groups (80% power at 5% alpha requires a sample size of 1350 patients).

Trial Registration Number

This study has been registered with ClinicalTrials.gov (identifier: NCT02662231). The registration is available to view via: https://clinicaltrials.gov/ct2/show/NCT02662231

For peer review only - http://bmjopen.bmj.com/site/about/guidelines.xhtml

Ethics and dissemination

Research ethics approval

The primary audit standard stems from the UK National Institute for Health and Clinical Excellence (NICE) guidelines [26] (Box 3). As this study will not change local clinical practice and is limited to using data obtained as part of usual care, it has been classified as an audit by the South East Scotland Research Ethics Service in Edinburgh, Scotland (Online Supplement 1). Thus, this may be considered a global audit or global service evaluation.

Investigators are required to gain approval from the relevant responsible bodies, including local Clinical Audit departments, Research and Development departments or Institutional Review Boards, as appropriate in their centre. If such departments are unavailable, written permission should be supplied by the Chief of Surgery or the responsible supervising consultant / attending physician.

Protocol Dissemination

The protocol will be disseminated across the established GlobalSurg network, compromised of surgeons, medical students and research nurses across the world. The network previously included over 1200 collaborators across 375 centres representing 58 countries [14]. Country leads are responsible for local co-ordination and dissemination within their country. In addition, the use of social media including Facebook, Twitter and YouTube has been shown to be an effective medium for dissemination of such collaborative projects [27] and will also be employed.

Dissemination of Results

We aim to publish the study results as open-access. Data from the study will be described to ensure individual countries, hospitals and surgeons are anonymous and then shall be deposited in an online data repository for others to analyse. Based on the results of GlobalSurg 2 Study, a quality improvement protocol and/or interventional clinical trials will be suggested for possible application in the infection control units of each hospital included in this study.

For peer review only - http://bmjopen.bmj.com/site/about/guidelines.xhtml

Discussion

In this study protocol, we describe a multicentre, international, prospective cohort study investigating the incidence of SSI and its association with preoperative parameters and risk factors as well as with post-operative morbidity and mortality. Despite SSI being the most common complication following major gastrointestinal surgery, no data exist describing its global epidemiology. By using collaborative methodology [1] and a short two-week data collection period, the study will recruit sufficient numbers of patients to inform this, while avoiding burdening low-resource centres that may otherwise be unable to participate. By investigating the morbidity and mortality caused by SSIs globally, this study will provide a platform to build future quality improvement programmes and interventional trials.

This study will be delivered using an international multi-disciplinary collaborative network of healthcare researchers. The collaborative model has consistently proven potential for producing high-quality outcomes data with limited resource requirements across national [8,19,28] and international studies [6]. A detailed study protocol and mandatory completion of online training modules will ensure standardised definitions and understanding of SSI so that this study will deliver a reliable and accurate data set. A pre-defined data validation strategy will produce an estimate of case ascertainment and the quality of local methods used to gather data.

SSIs have been shown to increase costs by an average of US$20,842 in high-income countries, which significantly burden health care systems [29]. Within low-income countries, health-care associated infections have been estimated to have an increased incidence [30]. This can result in an increased length of stay of an average 9.7 days longer and has further knock-on effects to dependants which are more profound in the low-income setting [29]. To help address this fiscal disparity, this project will inform future service-level quality improvement programmes. When regular audits of pathogens and antibiotic prescribing are undertaken, redundant antibiotic use is reduced [31,32]. With feedback of baseline SSI rates to participating centres, collaborators have the opportunity to appraise their current practice against a global standard. Surgeons will be able to implement interventions to improve service provision prior to a re-audit, one year in the future. With the savings made from the implementation of prevention programmes being 11 times greater on average than costs [33], these programmes will help reduce fiscal burden to patients and public health systems.

For peer review only - http://bmjopen.bmj.com/site/about/guidelines.xhtml

Many important determinants of a high burden of SSI in developing countries haven't been identified [7].This study will be able to identify an association between SSIs and various risk factors, however, due to its observational nature, will not be able to prove causality. Therefore, data points collected have been included with a plausible relationship with SSIs. The number of data collection points has been kept short in order to maximise data completeness and accuracy and minimise investigator workload.

Finally, this study will continue to strengthen the GlobalSurg network, developing capacity for research in low and middle-income countries[34]. For the many research-naive centres worldwide, participation in this project could instil a culture of clinical effectiveness into routine clinical practice. This study will ultimately provide the basis for a global multicentre randomised controlled trial, aimed at reducing the burden of SSI and its attributed morbidity and mortality.

For peer review only

References

1 Bhangu A, Kolias AG, Pinkney T, *et al.* Surgical research collaboratives in the UK. *Lancet* 2013;**382**:1091–2. doi:10.1016/S0140-6736(13)62013-9

2 Horan TC, Andrus M, Dudeck MA. CDC/NHSN surveillance definition of health care-associated infection and criteria for specific types of infections in the acute care setting. *Am J Infect Control* 2008;**36**:309–32. doi:10.1016/j.ajic.2008.03.002

3 Stewart B, Khanduri P, McCord C, *et al.* Global disease burden of conditions requiring emergency surgery. *Br J Surg* 2014;**101**:e9–22. doi:10.1002/bjs.9329

4 The Lancet Commission on Global Surgery. Data for the sustainable development of surgical systems: A global collaboration. 2015.http://www.lancetglobalsurgery.org/#!indicators/o217z (accessed 28 Dec2015).

5 Meara JG, Leather AJM, Hagander L, *et al.* Global Surgery 2030: evidence and solutions for achieving health, welfare, and economic development. *Lancet (London, England)* 2015;**386**:569–624. doi:10.1016/S0140-6736(15)60160-X

6 Pearse RM, Moreno RP, Bauer P, *et al.* Mortality after surgery in Europe: a 7 day cohort study. *Lancet (London, England)* 2012;**380**:1059–65. doi:10.1016/S0140-6736(12)61148-9

7 Allegranzi B, Nejad SB, Combescure C, *et al.* Burden of endemic health-care-associated infection in developing countries: Systematic review and meta-analysis. *Lancet* 2011;**377**:228–41. doi:10.1016/S0140-6736(10)61458-4

8 Pinkney TD, Calvert M, Bartlett DC, *et al.* Impact of wound edge protection devices on surgical site infection after laparotomy: multicentre randomised controlled trial (ROSSINI Trial). *BMJ* 2013;**347**:f4305.

9 Smyth ETM, McIlvenny G, Enstone JE, *et al.* Four country healthcare associated infection prevalence survey 2006: overview of the results. *J Hosp Infect* 2008;**69**:230–48. doi:10.1016/j.jhin.2008.04.020

10 Coello R, Charlett A, Wilson J, *et al.* Adverse impact of surgical site infections in English hospitals. *J Hosp Infect* 2005;**60**:93–103. doi:10.1016/j.jhin.2004.10.019

11 Wilson APR, Gibbons C, Reeves BC, *et al.* Surgical wound infection as a performance indicator: agreement of common definitions of wound infection in 4773 patients. *BMJ* 2004;**329**:720. doi:10.1136/bmj.38232.646227.DE

12 Plowman R, Graves N, Griffin MA, *et al.* The rate and cost of hospital-acquired

For peer review only - http://bmjopen.bmj.com/site/about/guidelines.xhtml

infections occurring in patients admitted to selected specialties of a district general hospital in England and the national burden imposed. *J Hosp Infect* 2001;**47**:198–209. doi:10.1053/jhin.2000.0881

13 Tanner J, Khan D, Aplin C, *et al.* Post-discharge surveillance to identify colorectal surgical site infection rates and related costs. *J Hosp Infect* 2009;**72**:243–50. doi:10.1016/j.jhin.2009.03.021

14 GlobalSurg Collaborative. GlobalSurg 1: Determining universal markers of quality in abdominal surgery: an international evaluation. 2015.http://globalsurg.org/projects/globalsurg-1/ (accessed 25 Dec2015).

15 Earnshaw S, Mendez A, Monnet DL, *et al.* Global collaboration to encourage prudent antibiotic use. *Lancet Infect Dis* 2013;**13**:1003–4. doi:10.1016/S1473-3099(13)70315-3

16 Dulon M, Haamann F, Peters C, *et al.* MRSA prevalence in European healthcare settings: a review. *BMC Infect Dis* 2011;**11**:138. doi:10.1186/1471-2334-11-138

17 Okeke IN, Laxminarayan R, Laxminarayan R, *et al.* Antimicrobial resistance in developing countries. Part I: recent trends and current status. *Lancet Infect Dis* 2005;**5**:481–93. doi:10.1016/S1473-3099(05)70189-4

18 Laxminarayan R, Duse A, Wattal C, *et al.* Antibiotic resistance-the need for global solutions. *Lancet Infect Dis* 2013;**13**:1057–98. doi:10.1016/S1473-3099(13)70318-9

19 STARSurg Collaborative. Impact of postoperative non-steroidal anti-inflammatory drugs on adverse events after gastrointestinal surgery. *Br J Surg* 2014;**101**:1413–23. doi:10.1002/bjs.9614

20 Bhangu A, Fitzgerald JE, Kolias AG. Trainee-led research collaboratives: a novel model for delivering multi-centre studies. *ANZ J Surg* 2014;**84**:902–3.http://www.ncbi.nlm.nih.gov/pubmed/25584384 (accessed 31 Jan2016).

21 Bhangu A, Fitzgerald JEF, Harrison EM. Global Academic Surgery and Global Outcomes in Surgery: Developing a Sustainable Model for the Development of Audit and Research Capacity. *JAMA Surg* 2016;**151**:94–5. doi:10.1001/jamasurg.2015.3082

22 Harris PA, Taylor R, Thielke R, *et al.* Research electronic data capture (REDCap)--a metadata-driven methodology and workflow process for providing translational research informatics support. *J Biomed Inform* 2009;**42**:377–81. doi:10.1016/j.jbi.2008.08.010

23 GlobalSurg Collaborative. GlobalSurg II Protocol | Globalsurg. 2015.http://globalsurg.org/globalsurg-2-protocol/ (accessed 29 Dec2015).

For peer review only - http://bmjopen.bmj.com/site/about/guidelines.xhtml

24 Programme UND. No Title. Hum. Dev. Index. 2014.http://hdr.undp.org/en/data.

25 GlobalSurg Collaborative. Mortalitly of emergency abdominal surgery in high, middle and low income countries. *Br J Surg* 2016.

26 England NHS, Groups CC. NICE support for commissioning for surgical site infection. 2013;:1–16.

27 Khatri C, Chapman SJ, Glasbey J, *et al.* Social media and internet driven study recruitment: evaluating a new model for promoting collaborator engagement and participation. *PLoS One* 2015;**10**:e0118899. doi:10.1371/journal.pone.0118899

28 Bhangu A. Safety of short, in-hospital delays before surgery for acute appendicitis: multicentre cohort study, systematic review, and meta-analysis. *Ann Surg* 2014;**259**:894–903. doi:10.1097/SLA.0000000000000492

29 de Lissovoy G, Fraeman K, Hutchins V, *et al.* Surgical site infection: Incidence and impact on hospital utilization and treatment costs. *Am J Infect Control* 2009;**37**:387–97. doi:10.1016/j.ajic.2008.12.010

30 Nejad SB, Allegranzi B, Syed S, *et al.* Health-care-associated infection in Africa: a systematic review. *Bull World Health Organ* 2011;**89**:757–65. doi:10.2471/BLT.11.088179

31 Cameron M, Jones S, Adedeji O. Antibiotic prophylaxis audit and questionnaire study: Traffic Light Poster improves adherence to protocol in gastrointestinal surgery. *Int J Surg* 2015;**19**:112–5. doi:10.1016/j.ijsu.2015.05.008

32 Ashiru-Oredope D, Sharland M, Charani E, *et al.* Improving the quality of antibiotic prescribing in the NHS by developing a new Antimicrobial Stewardship Programme: Start Smart--Then Focus. *J Antimicrob Chemother* 2012;**67 Suppl 1**:i51–63. doi:10.1093/jac/dks202

33 Arefian H, Vogel M, Kwetkat A, *et al.* Economic Evaluation of Interventions for Prevention of Hospital Acquired Infections: A Systematic Review. *PLoS One* 2016;**11**:e0146381. doi:10.1371/journal.pone.0146381

34 Spence RT, Panieri E, Rayne SL, *et al.* Collaboration is key to strengthening surgical research capacity in sub-Saharan Africa. *S Afr Med J* 2016;**106**:125–6.

For peer review only

For peer review only - http://bmjopen.bmj.com/site/about/guidelines.xhtml

Collaborators

Chetan Khatri, Midhun Mohan, Thomas M Drake, James Glasbey, Dmitri Nepogodiev, Catherine Shaw, Zahra Jaffry, Stuart Fergusson, Francesco Pata, Adesoji O Ademuyiwa, Afnan Altamini, Hosni Khairy Salem, Andrew Kirby, Kjetil Soreide, Gustavo Recinos, Richard Spence, Sarah Rayne, Stephen Tabiri, Jen Cornick, Thomas Pinkney, Richard Lilford, J Edward Fitzgerald, Ewen M Harrison, Aneel Bhangu

Author Contributions

All authors contributed to the design, drafting and review of this study protocol.

Competing Interests

The authors declare no competing interests with respect to this study. GlobalSurg is run by the Surgical Research Gateway (SuRG) Foundation. The SuRG Foundation is a registered charity (charity number 1 159 898) whose object is to advance the education of medical students and doctors in surgical science, clinical research and audit methods by promoting participation in collaborative clinical research and audit studies.

Footnotes

Twitter Follow @GlobalSurg https://twitter.com/GlobalSurg

For peer review only

Figures

Box 1: Patient inclusion and exclusion criteria

Inclusion criteria

- Patients of all ages (adult and paediatric)
- Consecutive patients during a chosen 14-day study period
- Undergoing emergency or elective gastrointestinal resection, cholecystectomy and appendicectomy
- Includes open, laparoscopic, laparoscopic converted and robotic cases
- Primary indication of trauma should be included
- Hernia repair with bowel resection should be included

Exclusion criteria

- Operations with a primary indication classified as vascular surgery, gynaecology, urology or transplantation
- Caesarean sections
- Whipples procedure
- Simple hernia repair

For peer review only

For peer review only - http://bmjopen.bmj.com/site/about/guidelines.xhtml

Box 2: CDC Criteria for SSI [2]

CDC criteria for SSI require the patient to have at least one of the following:

- Purulent drainage from the superficial or deep (fascia or muscle) incision but not from within the organ/space component of the surgical site
- At least one of the following: pain or tenderness; localised swelling; redness; heat; fever and the incision is opened deliberately or spontaneously dehisces
- Abscess within the wound (clinical, histopathological or radiological detection)

For peer review only

Box 3: Study Audit Standards [26]

Audit standards from National Institute for Health and Clinical Excellence:

Surveillance

- People having surgery are cared for by healthcare providers that monitor surgical site infection rates (including post-discharge infections) and provide feedback to relevant staff and stakeholders for continuous improvement through adjustment of clinical practice (Quality Statement 7).

Treatment of surgical site infection:

- People with a surgical site infection are offered treatment with an antibiotic that covers the likely causative organisms and is selected based on local resistance patterns and the results of microbiological tests (Quality Statement 6).

For peer review only

For peer review only - http://bmjopen.bmj.com/site/about/guidelines.xhtml

Table 1: Required Data Fields

Patient ID.	Local Hospital Field
Age	If >2 years = whole years, if <2 years = months
Gender	Male, Female
American Society of Anaesthesiologists (ASA) score	I, II, III, IV, V, not recorded
Immunosuppression	>Diabetes: Diet controlled, Tablet controlled, Insulin controlled, No<br>>HIV: Yes - on antiretroviral therapy; Yes - not on antiretroviral therapy, No, Unknown<br>>Steroids: Yes, No<br>>Other immunosuppressive drugs (e.g. azathioprine, methotrexate, biologic agents): Yes, No<br>>Chemotherapy (current chemotherapy or if the last cycle was within 12 weeks of operation): Yes, No<br>>Active malarial infection: Yes - confirmed by blood film or equivalent test, No
	HIV: *Yes* - most recent preoperative CD4 count
Smoking status	Current smoker (Including those who stopped smoking within the last 6 weeks), Previous smoker, Never smoked, Unknown
Date and time of admission	DD/MM/YYYY HH:MM
Date and time operation started (knife to skin time)	DD/MM/YYYY HH:MM
Length of operation (knife-to-skin until point of completion).	Minutes
Urgency of operation	Emergency (any surgery on the same admission as diagnosis), Semi-Elective, Elective (any planned admission for surgery)
Was a surgical safety checklist used (WHO or an equivalent)?	Yes, No - but available in this centre, No - not available in this centre
Initial operative approach	Open midline, Open non-midline, Laparoscopic, Laparoscopic converted to open, Robotic, Robotic converted to open
Primary operation performed	Pick from dropdown list; pick single main procedure performed.
	Appendicectomy - Appearance at surgery: simple (non-perforated), complex (perforated, free pus), normal
	Appendicectomy - Duration of symptoms (e.g. abdominal pain) prior to surgery (days, 0,1,2,3,4,5,6,7+)
Main surgical pathology/indication (The main cause leading to surgery).	Malignant (proven or suspected tumour/cancer), Benign
Intra-operative contamination	Clean Contaminated: GI tract entered but no gross contamination,<br>Contaminated: GI tract entered with gross spillage or major break in sterile technique,<br>Dirty: There is already contamination prior to operation (e.g. with faeces or bile).
**Antibiotic use:**	
Used for *treatment* before surgery (e.g. trial of antibiotics to treat diverticular abscess)	Yes (total days), No
Used for *prophylaxis* at the point of incision (i.e. standard hospital prophylaxis)	Yes, No
Continued at the end of surgery (i.e. extended prophylaxis after surgery)	Yes (total days), No
Was epidural analgesia inserted on the day of surgery?	Yes, No
Were NSAIDs used postoperatively during the first 5 days of after surgery? (Including ibuprofen, naproxen, diclofenac, ketorolac and etoricoxib, EXCLUDING aspirin).	Yes, No
Was serum haemoglobin/haematocrit checked in the first 48hrs postoperatively?	Yes - serum haemoglobin, Yes - capillary packed cell volume (PCV), No - but tests available in this centre, No - tests not available in this centre.
Was serum creatinine checked in the first 48hrs postoperatively?	Yes, No - but available in this centre, No - not available in this centre
Length of postoperative stay	Days
**Surgical site infection:**	
Prior to discharge	Yes, No
At 30 days after surgery	Yes, No, Not assessed after discharge

For peer review only

For peer review only - http://bmjopen.bmj.com/site/about/guidelines.xhtml

	If yes: Was a wound swab sent for microbiological culture: Yes, No - but available in this centre, No - not available in this centre
	If yes: How was this treated: operative drainage, wound opened outside of operating theatre, antibiotics (tick all that apply)
What bacteria, if any, were identified?	None, S. Aureus, Coliform, Anaerobe, Other (5 tick boxes).
	If yes: Sensitivity: sensitive to antibiotic prophylaxis given; resistant to antibiotic prophylaxis given; sensitivities not tested – but available in this centre; sensitivities not tested – not available in this centre
30-day unexpected re-intervention. Record the most serious re-intervention.	Yes - surgical, Yes - endoscopic, Yes - interventional radiology, No
30-day mortality	Dead, Alive, Unknown. *If died:* post-operative day of death
30-day intra-abdominal/pelvic abscess (CT, ultrasound, or clinical (including reoperation) evidence of intra-abdominal or pelvic abscess)	Yes, No
Other hospital acquired infection (Treated with or without antibiotics)	Yes - urinary tract infection, Yes - pneumonia, Yes - central venous line infection, Yes - peripheral line infection, Yes - other, No
How was 30-day follow-up status achieved? (All applicable)	Still an inpatient, Clinic review, Telephone review, Community/home review, Discharged before 30 days and not contacted again

For peer review only

For peer review only - http://bmjopen.bmj.com/site/about/guidelines.xhtml

South East Scotland Research Ethics Service

Waverley Gate
2-4 Waterloo Place
Edinburgh
EH1 3EG

NHS Lothian

Name: Stuart Fergusson
Address: 2/2 Boat Green
Edinburgh
EH3 5LL

Date: 16/10/2015
Your Ref:
Our Ref: NR/1510AB5
Enquiries to: Alex Bailey
Direct Line: 0131 465 5679
Email: alex.bailey@nhslothian.scot.nhs.uk

Dear Stuart,

Project Title: Determining the worldwide epidemiology of surgical site infections after gastrointestinal surgery

You have sought advice from the South East Scotland Research Ethics Service on the above project. This has been considered by the Scientific Officer and you are advised that, based on the submitted documentation (email correspondence and GS2 protocol v7_4_SJF), it does not need NHS ethical review under the terms of the Governance Arrangements for Research Ethics Committees (A Harmonised Edition).

The advice is based on the following:

- *The project is an audit limited to using data obtained as part of usual care, but note the requirement for Caldicott Guardian approval for the use or transfer of person-identifiable information within or from an organisation*

If the project is considered to be health-related research you will require a sponsor and ethical approval as outlined in The Research Governance Framework for Health and Community Care. You may wish to contact your employer or professional body to arrange this. You may also require NHS management permission (R&D approval). You should contact the relevant NHS R&D departments to organise this.

For projects that are not research and will be conducted within the NHS you should contact the relevant local clinical governance team who will inform you of the relevant governance procedures required before the project commences.

This letter should not be interpreted as giving a form of ethical approval or any endorsement of the project, but it may be provided to a journal or other body as evidence that NHS ethical approval is not required. However, if you, your sponsor/funder feel that the project requires ethical review by an NHS REC, please write setting out your reasons and we will be pleased to consider further. You should retain a copy of this letter with your project file as evidence that you have sought advice from the South East Scotland Research Ethics Service.

Yours sincerely,

Alex Bailey

Alex Bailey
Scientific Officer
South East Scotland Research Ethics Service

INVESTORS IN PEOPLE Healthy Working Lives

Headquarters
Waverley Gate, 2-4 Waterloo Place
Edinburgh EH1 3EG
Chair: Mr Brian Houston
Chief Executive: Tim Davison
Lothian NHS Board is the common name of Lothian Health Board

For peer review only

For peer review only - http://bmjopen.bmj.com/site/about/guidelines.xhtml

South East Scotland Research Ethics Service

Differentiating clinical audit, service evaluation, research and usual practice/surveillance work in public health

RESEARCH	SERVICE EVALUATION*	CLINICAL AUDIT	SURVEILLANCE	USUAL PRACTICE (in public health)
The attempt to derive generalizable new knowledge including studies that aim to generate hypotheses as well as studies that aim to test them.	Designed and conducted solely to define or judge current care.	Designed and conducted to produce information to inform delivery of best care.	Designed to manage outbreak and help the public by identifying and understanding risks associated.	Designed to investigate outbreak or incident to help in disease control and prevention.
Quantitative research – designed to test a hypothesis. Qualitative research – identifies/explores themes following established methodology.	Designed to answer: "What standard does this service achieve?"	Designed to answer: "Does this service reach a predetermined standard?"	Designed to answer: "What is the cause of this outbreak?"	Designed to answer: "What is the cause of this outbreak?" and treat.
Addresses clearly defined questions, aims and objectives.	Measures current service without reference to a standard.	Measures against a standard.	Systematic, statistical methods to allow timely public health action.	Systematic, statistical methods may be used.
Quantitative research – may involve evaluating or comparing interventions, particularly new ones. Qualitative research – usually involves studying how interventions and relationships are experienced.	Involves an intervention in use only. The choice of treatment is that of the clinician and patient according to guidance, professional standards and/or patient preference.	Involves an intervention in use only. The choice of treatment is that of the clinician and patient according to guidance, professional standards and/or patient preference.	May involve collecting personal data and samples with the intent to manage the incident.	Any choice of treatment is based on clinical best evidence or professional consensus.
Usually involves collecting data that are additional to those for routine care but may include data collected routinely. May involve treatments, samples or investigations additional to routine care.	Usually involves analysis of existing data but may include administration of interview or questionnaire.	Usually involves analysis of existing data but may include administration of simple interview or questionnaire.	May involve analysis of existing data or administration of interview or questionnaire to those exposed.	May involve administration of interview or questionnaire to those exposed.
Quantitative research – study design may involve allocating patients to intervention groups. Qualitative research – uses a clearly defined sampling framework underpinned by conceptual or theoretical justifications.	No allocation to intervention: the health professional and patient have chosen intervention before service evaluation.	No allocation to intervention: the health professional and patient have chosen intervention before audit.	Does not involve an intervention.	May involve allocation to control group to assess risk and identify source of incident but treatment unaffected.
May involve randomisation.	No randomisation.	No randomisation.	No randomisation.	May involve randomisation but not for treatment.
Normally requires REC review. Refer to www.nres.npsa.nhs.uk/applications/apply/ for more information.	Does not require REC review.	Does not require REC review.	Does not require REC review.	Does not require REC review.

* Service development and quality improvement may fall into this category.

Headquarters
Waverley Gate, 2-4 Waterloo Place
Edinburgh EH1 3EG
Chair: Mr Brian Houston
Chief Executive: Tim Davison
Lothian NHS Board is the common name of Lothian Health Board

For peer review only - http://bmjopen.bmj.com/site/about/guidelines.xhtml

BMJ Open

Determining the worldwide epidemiology of Surgical Site Infections after Gastrointestinal Resection Surgery: protocol for a multicentre, international, prospective cohort study (GlobalSurg 2).

Journal:	*BMJ Open*
Manuscript ID	bmjopen-2016-012150.R1
Article Type:	Protocol
Date Submitted by the Author:	22-Oct-2016
Complete List of Authors:	Khatri, Chetan<br>Collaborative, GlobalSurg<br>Bhangu, Aneel; University of Birmingham
**Primary Subject Heading**:	Surgery
Secondary Subject Heading:	Infectious diseases, Gastroenterology and hepatology, Global health
Keywords:	Globalsurgery, Surgical site Infection, Collaborative Research

SCHOLARONE™
Manuscripts

For peer review only

Determining the worldwide epidemiology of Surgical Site Infections after Gastrointestinal Resection Surgery: protocol for a multicentre, international, prospective cohort study (GlobalSurg 2).

Author: GlobalSurg Collaborative

***Correspondence*:**

Mr Aneel Bhangu MBChB MRCS PhD, Academic Department of Surgery
Room 29, 4th Floor, University of Birmingham, Birmingham, B15 2TH, UK
Email: aneelbhangu@doctors.org.uk
Telephone: 07789770619
Twiiter: @GlobalSurg

For peer review only

Declarations

Running title:	GlobalSurg 2 Project
Funding/Disclosure:	This project has received support from the British Medical Association and the Association of Surgeons in Training. These organisations have not been involved in the drafting of this protocol or review of this manuscript.
Abstract word count:	177
Word count:	2597 (excluding abstract, tables and references)
Key words:	Global Surgery, Surgical Site Infection, Collaborative Research

Authors' contributions: GlobalSurg Collaborative contributed significantly to study conception, protocol development, manuscript writing/editing and agreement for submission.

ABSTRACT

Introduction: Surgical site infection (SSI) is the most common complication following major gastrointestinal surgery, affecting between 25-40% of patients. The rate of SSI doubles from low-income to high-income settings, persisting after risk adjustment. The relative impact of antibiotic resistant organisms and the effectiveness of antibiotic prophylaxis globally are unknown. This study aims to determine SSI rates following gastrointestinal surgery across worldwide hospital settings.

Methods and analysis: This multicentre, international, prospective cohort study will be undertaken by any hospital providing emergency or elective gastroenterological surgical services. Centres will collect observational data on consecutive patients undergoing emergency or elective gastrointestinal resection, cholecystectomy or appendicectomy during a six-month period. The primary outcome is the incidence of SSI with secondary outcomes describing the organisms causing SSIs, including their antibiotic susceptibility, and the microbiological tests used to identify them.

Ethics and dissemination: This project will not affect clinical practice and has been classified as clinical audit following research ethics review. The protocol will be disseminated through the international GlobalSurg network.

Trial Registration Number: This study has been registered with ClinicalTrials.gov (identifier: NCT02662231).

For peer review only

For peer review only - http://bmjopen.bmj.com/site/about/guidelines.xhtml

Strengths and Limitations

- This will be the first international, multicentre, prospective study to assess the incidence of surgical site infections (SSI) for patients undergoing elective and emergency major gastrointestinal surgery.

- The collaborative methodology adopted, as described elsewhere [1], allows for large volume, high quality data collection whilst avoiding overburdening high-volume and low-resource centres that may otherwise be unable ot participate in such projects.

- Previous studies have had varying subjective definitions of SSI; this study uses standardised definitions from the Centre for Disease Control and Prevention (CDC) [2], which are widely implemented and internationally validated.

- Due to the observational nature of the study, it will not be possible to determine a causative link between risk factors and SSI and therefore comparisons of incidence and risk factors across differenct Human Develoment Index settings will be hypothesis generating only.

- As it is not possible to apply strict data monitoring within the confines of this study, we have developed a mixed-methods quantitative and qualitative validation process for our primary outcome measure, and case ascertainment rate, which will be applied to a sample of representative centres.

For peer review only - http://bmjopen.bmj.com/site/about/guidelines.xhtml

Introduction

The burden of surgical disease in low and middle-income countries (LMICs) is growing [3]. Specific programmes have aimed to highlight the unmet need for safe surgery and anaesthesia as part of the global health agenda [4]. The Lancet Commission on Global Surgery outlined six core indicators for the assessment of global surgical systems, including the postoperative mortality rate (POMR) [5]. While clearly important, mortality only affects 1-4% of all patients [6]. For major gastrointestinal surgery, quantifying POMR alone neglects the burden of postoperative morbidity, which affects a greater proportion of patients [4]. Other relevant markers of postoperative outcome are also needed to determine the success of surgery in the majority of patients who will survive.

Surgical site infection (SSI) is the most common complication following major gastrointestinal surgery [7], affecting between 25-40% of patients after midline laparotomy in high-income settings [8,9]. SSI is implicated in one-third of postoperative deaths and accounts for 8% of all deaths caused by a nosocomial infection [10–12]. Furthermore, SSIs cause pain and discomfort, increase hospital stay and put patients at greater risk of secondary infectious complications. This has an important economic impact with an attributable cost in the UK of £30 million per year [13].

Our 2014/15 prospective, observational cohort study (GlobalSurg 1 [14]) included 10,475 patients from 58 countries. It showed that the incidence of SSI more than doubled from high (7.4%), to middle (14.4%), to low (20.0%) income countries. This persisted after multivariable risk adjustment for patient and hospital confounders (middle income: odds ratio 1.96 [1.63-2.32] and low income: 2.06 [1.67-2.57]). In the most contaminated procedures, one in three patients from LMICs suffered an SSI. Dirty surgery doubled in low-income countries (29.7% versus 16.6% in high-income settings), which was in turn associated with doubling of SSI (34.5% low-income versus 15.4% high-income). However, SSI was assessed as a secondary outcome measure as part of that study, lacking validity and requiring external validation.

Antibiotic resistant organisms are now prevalent worldwide and a focus of interest for policy leaders and global health advocates [15]. In high-income settings, methicillin resistant *S. Aureus* accounts for up to 50% of *S. Aureus* infections [16]. While many hospitals in low income settings are not able to routinely perform surveillance of

For peer review only - http://bmjopen.bmj.com/site/about/guidelines.xhtml

resistant patterns, yet the little evidence that does exist suggests an accelerating incidence of antibiotic-resistant SSIs [17]. Patients with antibiotic resistant infections have a higher risk of mortality, morbidity and require more healthcare resources [18]. Currently, few data exist describing the international epidemiology of SSIs, the causative organisms or incidence of antimicrobial drug-resistance. Investigating the diagnosis and treatment of SSIs is an urgent global health priority, with important implications for patients, clinicians and policy makers.

Primary Aims

The primary aim is to determine the incidence of SSI following elective and emergency gastrointestinal surgery across low, middle and high Human Development Index (HDI) countries.

Secondary Aims

The secondary aims include describing the organisms causing SSIs, the incidence of antibiotic resistant pathogens and the microbiological tests used to identify them. The impact of the method of 30-day follow-up on these outcomes, where performed, will also be analysed.

For peer review only

Methods

Study Design

This is a multicentre, international, prospective, observational cohort study of all consecutive patients undergoing elective or emergency gastrointestinal surgery over a 14-day period. Individual collaborators are free to choose any 14-day period within the six-month study period to collect data. The study will run from the 4th of January 2016 to 31st July (with 30-day follow up of the last data collection period finishing on 30th August 2016). This 'snapshot' study design is a validated model that has been delivered successfully in previous studies [8,1,19].

The GlobalSurg Network

GlobalSurg (http://globalsurg.org/) is an international research collaborative, fostering regional, national and international surgical networks. The collaborative model used has previously been described elsewhere [20] and has already facilitated one multicentre, international, prospective cohort study [21]. GlobalSurg is run by the Surgical Research Gateway (SuRG) Foundation (http://surgfoundation.org/; registered charity number 1 159 898), in collaboration with the University of Birmingham and University of Edinburgh. The objective of the charity is to advance the education of medical students and doctors in surgical science, clinical research and audit methods by promoting participation in collaborative clinical research and audit studies.

Study Setting

Any surgical unit providing emergency or elective surgery worldwide is eligible to participate. Included centres must capture all consecutive patients and ensure data collection is >95% complete. Centres with >5% missing data, when including all possible data points, will be excluded from the final analysis and removed from authorship. The minimum number of patients that must be recruited is one. Multiple teams covering different non-overlapping time periods at each hospital are encouraged.

Patient inclusion and exclusion criteria

Patients of all ages (adult and paediatric) undergoing elective, semi-elective or emergency gastrointestinal resection, cholecystectomy or appendicectomy are eligible to enter (Box 1). Any operative approach can be used. Operations with a

For peer review only - http://bmjopen.bmj.com/site/about/guidelines.xhtml

primary indication classified as vascular surgery, gynaecology, urology or transplantation should be excluded.

Emergency procedures are defined as unplanned operations and include reoperations after previous procedures. Semi-elective procedures are defined as procedures planned on the background of the patient being discharged after an emergency admission in the past and is currently admitted for surgery. Gastrointestinal resection is defined as a complete transection and removal of a segment of the oesophagus, stomach, small bowel, colon or rectum.

Each individual patient should be included once into the study. Patients who return to theatre due to complications following earlier surgery can only be included if the index procedure is not already included.

Outcome measures

The primary outcome measure is the incidence of superficial or deep incisional SSI within 30 days of surgery involving any of the operative incisions made. This measure adopts the definitions within the 2008 Centre for Disease Control (CDC) [2] definitions of SSI (Box 2). Where it is unfeasible to follow up the patients after discharge for 30 days postoperatively, in-hospital SSI incidence will be used as a proxy measure.

The secondary outcomes that will be derived from this study include the:

1. A description of the global variation in organisms causing SSI (including use of microbiological tests).
2. The incidence of antibiotic resistant pathogens detected in patients with SSI.
3. 30-day post-operative mortality rate.
4. 30-day post-operative re-intervention rate.
5. To assess how difference in SSI rates vary based on the method by which patients are followed up.

Data points

Data points relating to patient, immunosuppression status, operative method and post-operative period will be collected (Table 1). In order to maximise data completeness, a minimal data set has been designed to test only factors relevant to the outcome measures. Investigators will enter data via the secure internet-based

For peer review only - http://bmjopen.bmj.com/site/about/guidelines.xhtml

For peer review only

Research Electronic Data Capture (REDCap) system [22]. Anonymous patient data will be held on the system hosted by the University of Edinburgh, Scotland, UK.

Investigators

The study will be undertaken by investigators around the world who will be responsible for disseminating the protocol at their individual site, ensuring appropriate study approvals are in place and collecting and uploading data to an online REDCap database. A central study writing committee compromising of an internationally representative group of health-care professionals will be responsible for data analysis, final manuscript drafting and submission. Individuals will be required to register their unit via the REDCap system and will be required to complete two training modules prior to starting.

Countries with multiple sites will be assigned a country lead, who will be responsible for co-ordinating multiple teams across sites to ensure duplication of data does not occur. Each hospital will have a primary local investigator who will act as guarantor of data. A maximum of three local investigators can cover each two week data collection period. They will be responsible for gaining local audit, service evaluation or research ethics approval as appropriate to their institution. Investigators should also create clear mechanisms to identify and include all eligible patients, which involves daily review of operating log books, emergency admission lists and team handover lists. They should identify clear pathways to accurately collect baseline and follow up data and be proactive in identifying postoperative events within the normal limits of follow-up.

Local arrangements for follow-up may include daily review of the patient and notes during admission and before discharge in order to identify in hospital SSIs, reviewing patient status in outpatient clinics or via telephone interview at 30 days (if this normal practice), checking hospital notes (paper or electronic) and looking at handover lists. All investigators will be listed as collaborators on resulting publications.

Quality of Data

To ensure high data quality, a detailed protocol has been produced and published online. The protocol has been translated into common languages to ease investigator understanding, including Chinese (Traditional and Simplified), French, Spanish,

For peer review only - http://bmjopen.bmj.com/site/about/guidelines.xhtml

Italian, Greek, Portuguese and Arabic [23]. In addition, investigators are mandated to complete two online educational modules, one a summary of the study protocol and the other an overview of the diagnosis and classification of surgical site infection.

Data Validation

Data validation will be performed in two parts across a group of representative centres. Case completeness assessment will involve an independent investigator ascertaining the number of eligible cases within a two-week data collection period at a participating centre and comparing this to actual number of cases submitted. Data accuracy will be assessed using qualitative, semi-structured interviews with investigators to assess local data collection methods.

Statistical analysis and power calculation

Identification of hospital or surgeon-specific performance will not be reported. Variation in outcome across different contexts will be tested using the Human Development Index (HDI) [24] (a composite statistic of life expectancy, education and income indices). Following analysis, centre-specific SSI rates will be fed back to participants. Hierarchical logistic regression multivariate analysis will be used to adjust the influence of HDI on SSI rates for confounding patient and disease variables. We will subgroup patients according to operative procedure for the purpose of analysis.

An appropriate sample size is difficult to calculate in this setting as there is a lack of previous study data to indicate the incidence of SSIs internationally. The previous GlobalSurg study recruited 10,745 patients using the same methodology [25].Although the inclusion criterion for this study is different, we expect to achieve a similar number of patients. Based on the findings from GlobalSurg 1, this number will provide adequate power to show a minimum difference from 7% to 10% in SSI rate between two groups (80% power at 5% alpha requires a sample size of 1350 patients).

Trial Registration Number

This study has been registered with ClinicalTrials.gov (identifier: NCT02662231). The registration is available to view via: https://clinicaltrials.gov/ct2/show/NCT02662231

For peer review only - http://bmjopen.bmj.com/site/about/guidelines.xhtml

Ethics and dissemination

Research ethics approval

The primary audit standard stems from the UK National Institute for Health and Clinical Excellence (NICE) guidelines [26] (Box 3). As this study will not change local clinical practice and is limited to using data obtained as part of usual care, it has been classified as an audit by the South East Scotland Research Ethics Service in Edinburgh, Scotland (Online Supplement 1). Thus, this may be considered a global audit or global service evaluation.

Investigators are required to gain approval from the relevant responsible bodies, including local Clinical Audit departments, Research and Development departments or Institutional Review Boards, as appropriate in their centre. If such departments are unavailable, written permission should be supplied by the Chief of Surgery or the responsible supervising consultant / attending physician.

Protocol Dissemination

The protocol will be disseminated across the established GlobalSurg network, compromised of surgeons, medical students and research nurses across the world. The network previously included over 1200 collaborators across 375 centres representing 58 countries [14]. Country leads are responsible for local co-ordination and dissemination within their country. In addition, the use of social media including Facebook, Twitter and YouTube has been shown to be an effective medium for dissemination of such collaborative projects [27] and will also be employed.

Dissemination of Results

We aim to publish the study results as open-access. Data from the study will be described to ensure individual countries, hospitals and surgeons are anonymous and then shall be deposited in an online data repository for others to analyse. Based on the results of GlobalSurg 2 Study, a quality improvement protocol and/or interventional clinical trials will be suggested for possible application in the infection control units of each hospital included in this study.

For peer review only

For peer review only - http://bmjopen.bmj.com/site/about/guidelines.xhtml

Discussion

In this study protocol, we describe a multicentre, international, prospective cohort study investigating the incidence of SSI and its association with preoperative parameters and risk factors as well as with post-operative morbidity and mortality. Despite SSI being the most common complication following major gastrointestinal surgery, no data exist describing its global epidemiology. Similarly, appendicetomies and cholecystectomies are two of the most common intra-abdominal operations performed; yet the international burden of SSI for these patients remains unknown.

By using collaborative methodology [1] and a short two-week data collection period, the study will recruit sufficient numbers of patients to inform this, while avoiding burdening low-resource centres that may otherwise be unable to participate. By investigating the morbidity and mortality caused by SSIs globally, this study will provide a platform to build future quality improvement programmes and interventional trials.

This study will be delivered using an international multi-disciplinary collaborative network of healthcare researchers. The collaborative model has consistently proven potential for producing high-quality outcomes data with limited resource requirements across national [8,19,28] and international studies [6]. A detailed study protocol and mandatory completion of online training modules will ensure standardised definitions and understanding of SSI so that this study will deliver a reliable and accurate data set. A pre-defined data validation strategy will produce an estimate of case ascertainment and the quality of local methods used to gather data.

SSIs have been shown to increase costs by an average of US$20,842 in high-income countries, which significantly burden health care systems [29]. Within low-income countries, health-care associated infections have been estimated to have an increased incidence [30]. This can result in an increased length of stay of an average 9.7 days longer and has further knock-on effects to dependants which are more profound in the low-income setting [29]. To help address this fiscal disparity, this project will inform future service-level quality improvement programmes. When regular audits of pathogens and antibiotic prescribing are undertaken, redundant antibiotic use is reduced [31,32]. With feedback of baseline SSI rates to participating centres, collaborators have the opportunity to appraise their current practice against a global standard. Surgeons will be able to implement interventions to improve

For peer review only - http://bmjopen.bmj.com/site/about/guidelines.xhtml

service provision prior to a re-audit, one year in the future. With the savings made from the implementation of prevention programmes being 11 times greater on average than costs [33], these programmes will help reduce fiscal burden to patients and public health systems.

Many important determinants of a high burden of SSI in developing countries haven't been identified [7].This study will be able to identify an association between SSIs and various risk factors, however, due to its observational nature, will not be able to prove causality. Therefore, data points collected have been included with a plausible relationship with SSIs. The number of data collection points has been kept short in order to maximise data completeness and accuracy and minimise investigator workload.

Finally, this study will continue to strengthen the GlobalSurg network, developing capacity for research in low and middle-income countries[34]. For the many research-naive centres worldwide, participation in this project could instil a culture of clinical effectiveness into routine clinical practice. This study will ultimately provide the basis for a global multicentre randomised controlled trial, aimed at reducing the burden of SSI and its attributed morbidity and mortality.

For peer review only

References

1 Bhangu A, Kolias AG, Pinkney T, *et al.* Surgical research collaboratives in the UK. *Lancet* 2013;**382**:1091–2. doi:10.1016/S0140-6736(13)62013-9

2 Horan TC, Andrus M, Dudeck MA. CDC/NHSN surveillance definition of health care-associated infection and criteria for specific types of infections in the acute care setting. *Am J Infect Control* 2008;**36**:309–32. doi:10.1016/j.ajic.2008.03.002

3 Stewart B, Khanduri P, McCord C, *et al.* Global disease burden of conditions requiring emergency surgery. *Br J Surg* 2014;**101**:e9–22. doi:10.1002/bjs.9329

4 The Lancet Commission on Global Surgery. Data for the sustainable development of surgical systems: A global collaboration. 2015.http://www.lancetglobalsurgery.org/#!indicators/o217z (accessed 28 Dec2015).

5 Meara JG, Leather AJM, Hagander L, *et al.* Global Surgery 2030: evidence and solutions for achieving health, welfare, and economic development. *Lancet (London, England)* 2015;**386**:569–624. doi:10.1016/S0140-6736(15)60160-X

6 Pearse RM, Moreno RP, Bauer P, *et al.* Mortality after surgery in Europe: a 7 day cohort study. *Lancet (London, England)* 2012;**380**:1059–65. doi:10.1016/S0140-6736(12)61148-9

7 Allegranzi B, Nejad SB, Combescure C, *et al.* Burden of endemic health-care-associated infection in developing countries: Systematic review and meta-analysis. *Lancet* 2011;**377**:228–41. doi:10.1016/S0140-6736(10)61458-4

8 Pinkney TD, Calvert M, Bartlett DC, *et al.* Impact of wound edge protection devices on surgical site infection after laparotomy: multicentre randomised controlled trial (ROSSINI Trial). *BMJ* 2013;**347**:f4305.

9 Smyth ETM, McIlvenny G, Enstone JE, *et al.* Four country healthcare associated infection prevalence survey 2006: overview of the results. *J Hosp Infect* 2008;**69**:230–48. doi:10.1016/j.jhin.2008.04.020

10 Coello R, Charlett A, Wilson J, *et al.* Adverse impact of surgical site infections in English hospitals. *J Hosp Infect* 2005;**60**:93–103. doi:10.1016/j.jhin.2004.10.019

11 Wilson APR, Gibbons C, Reeves BC, *et al.* Surgical wound infection as a performance indicator: agreement of common definitions of wound infection in 4773 patients. *BMJ* 2004;**329**:720. doi:10.1136/bmj.38232.646227.DE

12 Plowman R, Graves N, Griffin MA, *et al.* The rate and cost of hospital-acquired

For peer review only

infections occurring in patients admitted to selected specialties of a district general hospital in England and the national burden imposed. *J Hosp Infect* 2001;**47**:198–209. doi:10.1053/jhin.2000.0881

13 Tanner J, Khan D, Aplin C, *et al.* Post-discharge surveillance to identify colorectal surgical site infection rates and related costs. *J Hosp Infect* 2009;**72**:243–50. doi:10.1016/j.jhin.2009.03.021

14 GlobalSurg Collaborative. GlobalSurg 1: Determining universal markers of quality in abdominal surgery: an international evaluation. 2015.http://globalsurg.org/projects/globalsurg-1/ (accessed 25 Dec2015).

15 Earnshaw S, Mendez A, Monnet DL, *et al.* Global collaboration to encourage prudent antibiotic use. *Lancet Infect Dis* 2013;**13**:1003–4. doi:10.1016/S1473-3099(13)70315-3

16 Dulon M, Haamann F, Peters C, *et al.* MRSA prevalence in European healthcare settings: a review. *BMC Infect Dis* 2011;**11**:138. doi:10.1186/1471-2334-11-138

17 Okeke IN, Laxminarayan R, Laxminarayan R, *et al.* Antimicrobial resistance in developing countries. Part I: recent trends and current status. *Lancet Infect Dis* 2005;**5**:481–93. doi:10.1016/S1473-3099(05)70189-4

18 Laxminarayan R, Duse A, Wattal C, *et al.* Antibiotic resistance-the need for global solutions. *Lancet Infect Dis* 2013;**13**:1057–98. doi:10.1016/S1473-3099(13)70318-9

19 STARSurg Collaborative. Impact of postoperative non-steroidal anti-inflammatory drugs on adverse events after gastrointestinal surgery. *Br J Surg* 2014;**101**:1413–23. doi:10.1002/bjs.9614

20 Bhangu A, Fitzgerald JE, Kolias AG. Trainee-led research collaboratives: a novel model for delivering multi-centre studies. *ANZ J Surg* 2014;**84**:902–3.http://www.ncbi.nlm.nih.gov/pubmed/25584384 (accessed 31 Jan2016).

21 Bhangu A, Fitzgerald JEF, Harrison EM. Global Academic Surgery and Global Outcomes in Surgery: Developing a Sustainable Model for the Development of Audit and Research Capacity. *JAMA Surg* 2016;**151**:94–5. doi:10.1001/jamasurg.2015.3082

22 Harris PA, Taylor R, Thielke R, *et al.* Research electronic data capture (REDCap)--a metadata-driven methodology and workflow process for providing translational research informatics support. *J Biomed Inform* 2009;**42**:377–81. doi:10.1016/j.jbi.2008.08.010

23 GlobalSurg Collaborative. GlobalSurg II Protocol | Globalsurg. 2015.http://globalsurg.org/globalsurg-2-protocol/ (accessed 29 Dec2015).

For peer review only - http://bmjopen.bmj.com/site/about/guidelines.xhtml

24 Programme UND. No Title. Hum. Dev. Index. 2014.http://hdr.undp.org/en/data.
25 GlobalSurg Collaborative. Mortalitly of emergency abdominal surgery in high, middle and low income countries. *Br J Surg* 2016.
26 England NHS, Groups CC. NICE support for commissioning for surgical site infection. 2013;:1–16.
27 Khatri C, Chapman SJ, Glasbey J, *et al.* Social media and internet driven study recruitment: evaluating a new model for promoting collaborator engagement and participation. *PLoS One* 2015;**10**:e0118899. doi:10.1371/journal.pone.0118899
28 Bhangu A. Safety of short, in-hospital delays before surgery for acute appendicitis: multicentre cohort study, systematic review, and meta-analysis. *Ann Surg* 2014;**259**:894–903. doi:10.1097/SLA.0000000000000492
29 de Lissovoy G, Fraeman K, Hutchins V, *et al.* Surgical site infection: Incidence and impact on hospital utilization and treatment costs. *Am J Infect Control* 2009;**37**:387–97. doi:10.1016/j.ajic.2008.12.010
30 Nejad SB, Allegranzi B, Syed S, *et al.* Health-care-associated infection in Africa: a systematic review. *Bull World Health Organ* 2011;**89**:757–65. doi:10.2471/BLT.11.088179
31 Cameron M, Jones S, Adedeji O. Antibiotic prophylaxis audit and questionnaire study: Traffic Light Poster improves adherence to protocol in gastrointestinal surgery. *Int J Surg* 2015;**19**:112–5. doi:10.1016/j.ijsu.2015.05.008
32 Ashiru-Oredope D, Sharland M, Charani E, *et al.* Improving the quality of antibiotic prescribing in the NHS by developing a new Antimicrobial Stewardship Programme: Start Smart--Then Focus. *J Antimicrob Chemother* 2012;**67 Suppl 1**:i51–63. doi:10.1093/jac/dks202
33 Arefian H, Vogel M, Kwetkat A, *et al.* Economic Evaluation of Interventions for Prevention of Hospital Acquired Infections: A Systematic Review. *PLoS One* 2016;**11**:e0146381. doi:10.1371/journal.pone.0146381
34 Spence RT, Panieri E, Rayne SL, *et al.* Collaboration is key to strengthening surgical research capacity in sub-Saharan Africa. *S Afr Med J* 2016;**106**:125–6.

For peer review only - http://bmjopen.bmj.com/site/about/guidelines.xhtml

Collaborators

Chetan Khatri, Midhun Mohan, Thomas M Drake, James Glasbey, Dmitri Nepogodiev, Catherine Shaw, Zahra Jaffry, Stuart Fergusson, Francesco Pata, Adesoji O Ademuyiwa, Afnan Altamini, Hosni Khairy Salem, Andrew Kirby, Kjetil Soreide, Gustavo Recinos, Richard Spence, Sarah Rayne, Stephen Tabiri, Jen Cornick, Thomas Pinkney, Richard Lilford, J Edward Fitzgerald, Ewen M Harrison, Aneel Bhangu

Author Contributions

All collaborators (CK, MM, TD, JG, DN, CS, ZJ, SF, FP, AOA, AA, HKS, AK, KS, GR, RS, SR, ST, JC, TP, RL, JEF, EMH, AB) contributed significantly to study conception, protocol development, manuscript writing/editing and agreement for submission.

Competing Interests

The authors declare no competing interests with respect to this study. GlobalSurg is run by the Surgical Research Gateway (SuRG) Foundation. The SuRG Foundation is a registered charity (charity number 1 159 898) whose object is to advance the education of medical students and doctors in surgical science, clinical research and audit methods by promoting participation in collaborative clinical research and audit studies.

Footnotes

Twitter Follow @GlobalSurg https://twitter.com/GlobalSurg

For peer review only

Figures

Box 1: Patient inclusion and exclusion criteria

Inclusion criteria

- Patients of all ages (adult and paediatric)
- Consecutive patients during a chosen 14-day study period
- Undergoing emergency or elective gastrointestinal resection, cholecystectomy and appendicectomy
- Includes open, laparoscopic, laparoscopic converted and robotic cases
- Primary indication of trauma should be included
- Hernia repair with bowel resection should be included

Exclusion criteria

- Operations with a primary indication classified as vascular surgery, gynaecology, urology or transplantation
- Caesarean sections
- Whipples procedure
- Simple hernia repair

For peer review only

For peer review only - http://bmjopen.bmj.com/site/about/guidelines.xhtml

Box 2: CDC Criteria for SSI [2]

CDC criteria for SSI require the patient to have at least one of the following:

- Purulent drainage from the superficial or deep (fascia or muscle) incision but not from within the organ/space component of the surgical site
- At least one of the following: pain or tenderness; localised swelling; redness; heat; fever and the incision is opened deliberately or spontaneously dehisces
- Abscess within the wound (clinical, histopathological or radiological detection)

For peer review only

For peer review only - http://bmjopen.bmj.com/site/about/guidelines.xhtml

Box 3: Study Audit Standards [26]

Audit standards from National Institute for Health and Clinical Excellence:

Surveillance

- People having surgery are cared for by healthcare providers that monitor surgical site infection rates (including post-discharge infections) and provide feedback to relevant staff and stakeholders for continuous improvement through adjustment of clinical practice (Quality Statement 7).

Treatment of surgical site infection:

- People with a surgical site infection are offered treatment with an antibiotic that covers the likely causative organisms and is selected based on local resistance patterns and the results of microbiological tests (Quality Statement 6).

For peer review only

For peer review only - http://bmjopen.bmj.com/site/about/guidelines.xhtml

Table 1: Required Data Fields

Patient ID.	Local Hospital Field
Age	If >2 years = whole years, if <2 years = months
Gender	Male, Female
American Society of Anaesthesiologists (ASA) score	I, II, III, IV, V, not recorded
Immunosuppression	>Diabetes: Diet controlled, Tablet controlled, Insulin controlled, No<br>>HIV: Yes - on antiretroviral therapy; Yes - not on antiretroviral therapy, No, Unknown<br>>Steroids: Yes, No<br>>Other immunosuppressive drugs (e.g. azathioprine, methotrexate, biologic agents): Yes, No<br>>Chemotherapy (current chemotherapy or if the last cycle was within 12 weeks of operation): Yes, No<br>>Active malarial infection: Yes - confirmed by blood film or equivalent test, No
	HIV: *Yes* - most recent preoperative CD4 count
Smoking status	Current smoker (Including those who stopped smoking within the last 6 weeks), Previous smoker, Never smoked, Unknown
Date and time of admission	DD/MM/YYYY HH:MM
Date and time operation started (knife to skin time)	DD/MM/YYYY HH:MM
Length of operation (knife-to-skin until point of completion).	Minutes
Urgency of operation	Emergency (any surgery on the same admission as diagnosis), Semi-Elective, Elective (any planned admission for surgery)
Was a surgical safety checklist used (WHO or an equivalent)?	Yes, No - but available in this centre, No - not available in this centre
Initial operative approach	Open midline, Open non-midline, Laparoscopic, Laparoscopic converted to open, Robotic, Robotic converted to open
Primary operation performed	Pick from dropdown list; pick single main procedure performed.
	Appendicectomy - Appearance at surgery: simple (non-perforated), complex (perforated, free pus), normal
	Appendicectomy - Duration of symptoms (e.g. abdominal pain) prior to surgery (days, 0,1,2,3,4,5,6,7+)
Main surgical pathology/indication (The main cause leading to surgery).	Malignant (proven or suspected tumour/cancer), Benign
Intra-operative contamination	Clean Contaminated: GI tract entered but no gross contamination,<br>Contaminated: GI tract entered with gross spillage or major break in sterile technique,<br>Dirty: There is already contamination prior to operation (e.g. with faeces or bile).
**Antibiotic use:**	
Used for *treatment* before surgery (e.g. trial of antibiotics to treat diverticular abscess)	Yes (total days), No
Used for *prophylaxis* at the point of incision (i.e. standard hospital prophylaxis)	Yes, No
Continued at the end of surgery (i.e. extended prophylaxis after surgery)	Yes (total days), No
Was epidural analgesia inserted on the day of surgery?	Yes, No
Were NSAIDs used postoperatively during the first 5 days of after surgery? (Including ibuprofen, naproxen, diclofenac, ketorolac and etoricoxib, EXCLUDING aspirin).	Yes, No
Was serum haemoglobin/haematocrit checked in the first 48hrs postoperatively?	Yes - serum haemoglobin, Yes - capillary packed cell volume (PCV), No - but tests available in this centre, No - tests not available in this centre.
Was serum creatinine checked in the first 48hrs postoperatively?	Yes, No - but available in this centre, No - not available in this centre
Length of postoperative stay	Days
**Surgical site infection:**	
Prior to discharge	Yes, No
At 30 days after surgery	Yes, No, Not assessed after discharge

For peer review only

For peer review only - http://bmjopen.bmj.com/site/about/guidelines.xhtml

	If yes: Was a wound swab sent for microbiological culture: Yes, No - but available in this centre, No - not available in this centre
	If yes: How was this treated: operative drainage, wound opened outside of operating theatre, antibiotics (tick all that apply)
What bacteria, if any, were identified?	None, S. Aureus, Coliform, Anaerobe, Other (5 tick boxes).
	If yes: Sensitivity: sensitive to antibiotic prophylaxis given; resistant to antibiotic prophylaxis given; sensitivities not tested – but available in this centre; sensitivities not tested – not available in this centre
30-day unexpected re-intervention. Record the most serious re-intervention.	Yes - surgical, Yes - endoscopic, Yes - interventional radiology, No
30-day mortality	Dead, Alive, Unknown. *If died:* post-operative day of death
30-day intra-abdominal/pelvic abscess (CT, ultrasound, or clinical (including reoperation) evidence of intra-abdominal or pelvic abscess)	Yes, No
Other hospital acquired infection (Treated with or without antibiotics)	Yes - urinary tract infection, Yes - pneumonia, Yes - central venous line infection, Yes - peripheral line infection, Yes - other, No
How was 30-day follow-up status achieved? (All applicable)	Still an inpatient, Clinic review, Telephone review, Community/home review, Discharged before 30 days and not contacted again

For peer review only

South East Scotland Research Ethics Service

Waverley Gate
2-4 Waterloo Place
Edinburgh
EH1 3EG

NHS Lothian

Name: Stuart Fergusson
Address: 2/2 Boat Green
Edinburgh
EH3 5LL

Date: 16/10/2015
Your Ref:
Our Ref: NR/1510AB5
Enquiries to: Alex Bailey
Direct Line: 0131 465 5679
Email: alex.bailey@nhslothian.scot.nhs.uk

Dear Stuart,

Project Title: Determining the worldwide epidemiology of surgical site infections after gastrointestinal surgery

You have sought advice from the South East Scotland Research Ethics Service on the above project. This has been considered by the Scientific Officer and you are advised that, based on the submitted documentation (email correspondence and GS2 protocol v7_4_SJF), it does not need NHS ethical review under the terms of the Governance Arrangements for Research Ethics Committees (A Harmonised Edition).

The advice is based on the following:

- *The project is an audit limited to using data obtained as part of usual care, but note the requirement for Caldicott Guardian approval for the use or transfer of person-identifiable information within or from an organisation*

If the project is considered to be health-related research you will require a sponsor and ethical approval as outlined in The Research Governance Framework for Health and Community Care. You may wish to contact your employer or professional body to arrange this. You may also require NHS management permission (R&D approval). You should contact the relevant NHS R&D departments to organise this.

For projects that are not research and will be conducted within the NHS you should contact the relevant local clinical governance team who will inform you of the relevant governance procedures required before the project commences.

This letter should not be interpreted as giving a form of ethical approval or any endorsement of the project, but it may be provided to a journal or other body as evidence that NHS ethical approval is not required. However, if you, your sponsor/funder feel that the project requires ethical review by an NHS REC, please write setting out your reasons and we will be pleased to consider further. You should retain a copy of this letter with your project file as evidence that you have sought advice from the South East Scotland Research Ethics Service.

Yours sincerely,

Alex Bailey

Alex Bailey
Scientific Officer
South East Scotland Research Ethics Service

INVESTORS IN PEOPLE
POSITIVE ABOUT DISABLED PEOPLE
Healthy Working Lives

Headquarters
Waverley Gate, 2-4 Waterloo Place
Edinburgh EH1 3EG
Chair: Mr Brian Houston
Chief Executive: Tim Davison
Lothian NHS Board is the common name of Lothian Health Board

For peer review only - http://bmjopen.bmj.com/site/about/guidelines.xhtml

For peer review only

South East Scotland Research Ethics Service

Differentiating clinical audit, service evaluation, research and usual practice/surveillance work in public health

RESEARCH	SERVICE EVALUATION*	CLINICAL AUDIT	SURVEILLANCE	USUAL PRACTICE (in public health)
The attempt to derive generalizable new knowledge including studies that aim to generate hypotheses as well as studies that aim to test them.	Designed and conducted solely to define or judge current care.	Designed and conducted to produce information to inform delivery of best care.	Designed to manage outbreak and help the public by identifying and understanding risks associated.	Designed to investigate outbreak or incident to help in disease control and prevention.
Quantitative research – designed to test a hypothesis. Qualitative research – identifies/explores themes following established methodology.	Designed to answer: "What standard does this service achieve?"	Designed to answer: "Does this service reach a predetermined standard?"	Designed to answer: "What is the cause of this outbreak?"	Designed to answer: "What is the cause of this outbreak?" and treat.
Addresses clearly defined questions, aims and objectives.	Measures current service without reference to a standard.	Measures against a standard.	Systematic, statistical methods to allow timely public health action.	Systematic, statistical methods may be used.
Quantitative research – may involve evaluating or comparing interventions, particularly new ones. Qualitative research – usually involves studying how interventions and relationships are experienced.	Involves an intervention in use only. The choice of treatment is that of the clinician and patient according to guidance, professional standards and/or patient preference.	Involves an intervention in use only. The choice of treatment is that of the clinician and patient according to guidance, professional standards and/or patient preference.	May involve collecting personal data and samples with the intent to manage the incident.	Any choice of treatment is based on clinical best evidence or professional consensus.
Usually involves collecting data that are additional to those for routine care but may include data collected routinely. May involve treatments, samples or investigations additional to routine care.	Usually involves analysis of existing data but may include administration of interview or questionnaire.	Usually involves analysis of existing data but may include administration of simple interview or questionnaire.	May involve analysis of existing data or administration of interview or questionnaire to those exposed.	May involve administration of interview or questionnaire to those exposed.
Quantitative research – study design may involve allocating patients to intervention groups. Qualitative research – uses a clearly defined sampling framework underpinned by conceptual or theoretical justifications.	No allocation to intervention: the health professional and patient have chosen intervention before service evaluation.	No allocation to intervention: the health professional and patient have chosen intervention before audit.	Does not involve an intervention.	May involve allocation to control group to assess risk and identify source of incident but treatment unaffected.
May involve randomisation.	No randomisation.	No randomisation.	No randomisation.	May involve randomisation but not for treatment.
Normally requires REC review. Refer to www.nres.npsa.nhs.uk/applications/apply/ for more information.	Does not require REC review.	Does not require REC review.	Does not require REC review.	Does not require REC review.

* Service development and quality improvement may fall into this category.

Headquarters
Waverley Gate, 2-4 Waterloo Place
Edinburgh EH1 3EG
Chair: Mr Brian Houston
Chief Executive: Tim Davison
Lothian NHS Board is the common name of Lothian Health Board

For peer review only - http://bmjopen.bmj.com/site/about/guidelines.xhtml
